# Intelligent Luminance Control of Lighting Systems Based on Imaging Sensor Feedback

**DOI:** 10.3390/s17020321

**Published:** 2017-02-09

**Authors:** Haoting Liu, Qianxiang Zhou, Jin Yang, Ting Jiang, Zhizhen Liu, Jie Li

**Affiliations:** 1 School of Biological Science and Medical Engineering, Beihang University, Beijing 100191, China; imkyran@hotmail.com; 2 Astronaut Research and Training Center of China, Beijing 100094, China; yangj_acc@sina.com (J.Y.); jiangt_acc@sina.com (T.J.); liuzz_acc@sina.com (Z.L.); lijie_nudt@126.com (J.L.)

**Keywords:** intelligent lighting, luminance control, vision effect, environment perception, imaging sensor

## Abstract

An imaging sensor-based intelligent Light Emitting Diode (LED) lighting system for desk use is proposed. In contrast to the traditional intelligent lighting system, such as the photosensitive resistance sensor-based or the infrared sensor-based system, the imaging sensor can realize a finer perception of the environmental light; thus it can guide a more precise lighting control. Before this system works, first lots of typical imaging lighting data of the desk application are accumulated. Second, a series of subjective and objective Lighting Effect Evaluation Metrics (LEEMs) are defined and assessed for these datasets above. Then the cluster benchmarks of these objective LEEMs can be obtained. Third, both a single LEEM-based control and a multiple LEEMs-based control are developed to realize a kind of optimal luminance tuning. When this system works, first it captures the lighting image using a wearable camera. Then it computes the objective LEEMs of the captured image and compares them with the cluster benchmarks of the objective LEEMs. Finally, the single LEEM-based or the multiple LEEMs-based control can be implemented to get a kind of optimal lighting effect. Many experiment results have shown the proposed system can tune the LED lamp automatically according to environment luminance changes.

## 1. Introduction

**Figure 1:**
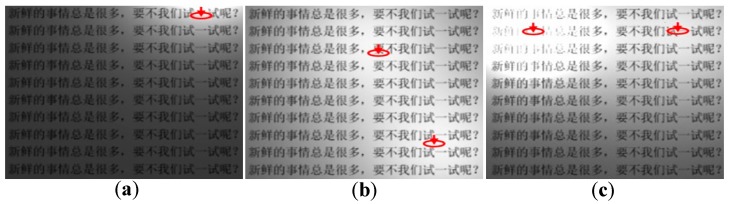
The different lighting effects of a display: (**a**) The display lighting effect of a sole light source; (**b**) the display lighting effect of multiple light sources; (**c**) an improper display lighting effect with glare.

In recent years intelligent Light Emitting Diode (LED) lighting systems have been widely applied in our daily life, such as in intelligent street lighting systems [1,2], intelligent office lighting devices, or even the tele-robotic arm equipment [3]. The intelligent lighting system uses a mini-type sensor and its control circuit to perceive the environment luminance, then estimates the lighting effect, and implements a precise output control of LED lamp(s) [4]; then an optimal lighting vision effect can be obtained. The intelligent lighting system is not used only in the nighttime; it also can be employed as a light compensation tool to solve complex environment light source problems or uneven illumination distribution problems during the daytime. Improper lighting will decrease working efficiency [5], affect task scheduling, or even cause some accidents. For example, in our past research work it can be found that the visual fatigue and the human error rate may be increased when an astronaut participates in some aerospace flight tasks under improper environment lighting [6]. Figure 1 shows different lighting effects of a display, where: (a) uses one light source; (b) and (c) use two light sources, and (c) suffers from the glare. From Figure 1, it can be found that different lighting effects will seriously influence the display reading task [7]. Thus it is necessary to develop an intelligent lighting system and method to achieve optimal lighting effects.

Many research works have been done to improve the output performance of intelligent LED lighting systems. For example, in [8], the authors developed a system which utilized a passive infrared sensor to control street lamps according to vehicle detection results. In [9], the authors used an infrared sensor to detect human movement, and a photosensitive resistance was also employed to tune the lighting intensity. In [10], the authors used an ultrasonic array sensor to detect the indoor presence of people so that an energy-efficient occupancy-adaptive indoor lighting system was realized. Obviously, the traditional sensors, such as the infrared sensor, the photosensitive resistance sensor, or the ultrasonic sensor, only have limited environment perception ability. Their outputs are too “coarse” for the lighting effect analysis because they only can acquire one-dimensional sensor data or they just provide a switching value. Thus, to get an elaborate description and understanding of the external environment, other sensors should be considered.

The aim of this research work is to use an imaging sensor to perceive environmental luminance changes and implement a kind of intelligent lighting control. Without loss of generality, let us take an indoor desk application as a research object. Before this system is employed, first lots of imaging lighting data of the desk application are accumulated. A wearable camera can be used to collect images. Second, a series of subjective and objective Lighting Effect Evaluation Metrics (LEEMs) are defined and evaluated for these datasets above; and then the cluster benchmarks of these objective LEEMs can be obtained. The LEEMs include: the Subjective and the Objective Surface Luminance Degree [11] of Display *SM_SLDD_* and *OM_SLDD_*, the Subjective and the Objective Color Deviation Degree [12] of Display Content *SM_CDDDC_* and *OM_CDDDC_*, the Subjective and the Objective Region Contrast Degree [13] of Display Content *SM_RCDDC_* and *OM_RCDDC_*, the Subjective and the Objective Edge Blur Degree [14] of Display Content *SM_EBDDC_* and *OM_EBDDC_*, and the Objective Glare Degree [15] of Display *OM_GDD_*. Third, both a single LEEM-based control and a multiple LEEMs-based control are developed to realize a kind of optimal luminance tuning. When this system works, first it captures the lighting image by a wearable camera. Then it computes the objective LEEMs of the captured image data above and compares the corresponding results with the cluster benchmarks of the objective LEEMs. Finally, the single LEEM-based or the multiple LEEMs-based control can be implemented to get an optimal lighting effect.

The main contributions of this paper include: first, an imaging environment perception method of lighting effects is developed. A series of blind image quality evaluation metrics [16] are utilized as the perception features; thus the lighting effect can be described and controlled quantitatively. Second, a practical application system of proposed technique is designed and developed. The wearable camera sensor together with a color card and a LED lamp are considered in this system; the intelligent hardware product is also employed to realize the corresponding information processing function. In the following sections, first a big view of proposed system is presented. Then the specific techniques are introduced. Finally, some experiment results and discussions are given.

## 2. Proposed Lighting System Design

### 2.1. Application Formulations

**Figure 2:**
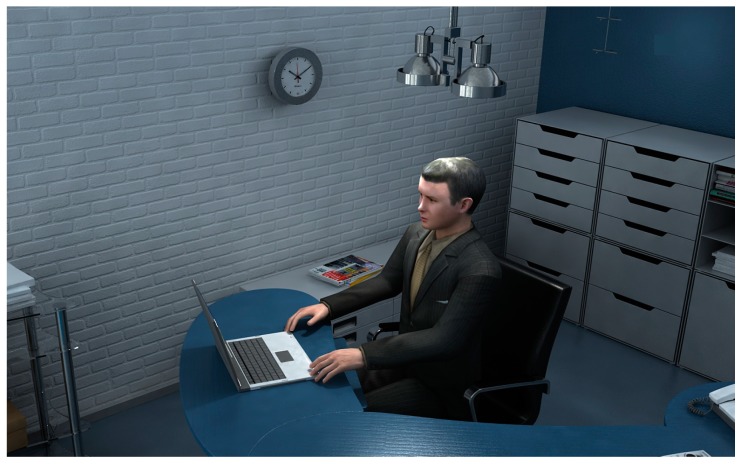
The application prototype of proposed lighting system.

The prototype of the proposed intelligent LED lighting system for desk applications is shown in Figure 2. This system works only for a single user. This prototype has many application instances, such as the hermetic cockpit design for pilots or industrial worktable development for workers, etc. In Figure 2, a user sits in front of a display to perform a reading or writing task; a white light LED source is fixed in the ceiling above the user’s head; the distance between the LED lighting source and the user’s head is from 0.25 m to 1.0 m. A surface light source is utilized here. The output intensity of LED lamp can be adaptively tuned by its controller. This proposed lighting system can work for both daytime and nighttime application. If it works for a daytime application, this LED lamp can be used as a lighting supplement apparatus: it can balance the uneven luminance effect for an ocular operation task. Differently, if it is utilized for a nighttime application, it can work as a lighting providing source. In this paper, it is supposed that the size of display is about 200 mm × 300 mm; its luminance output is stable. After an extensive investigation of the application requirements, here the “good lighting effect” means the normal color and a proper environment luminance (including the intensity, the contrast, and the distribution) without any glare.

### 2.2. Design of Intelligent LED Lighting System

**Figure 3:**
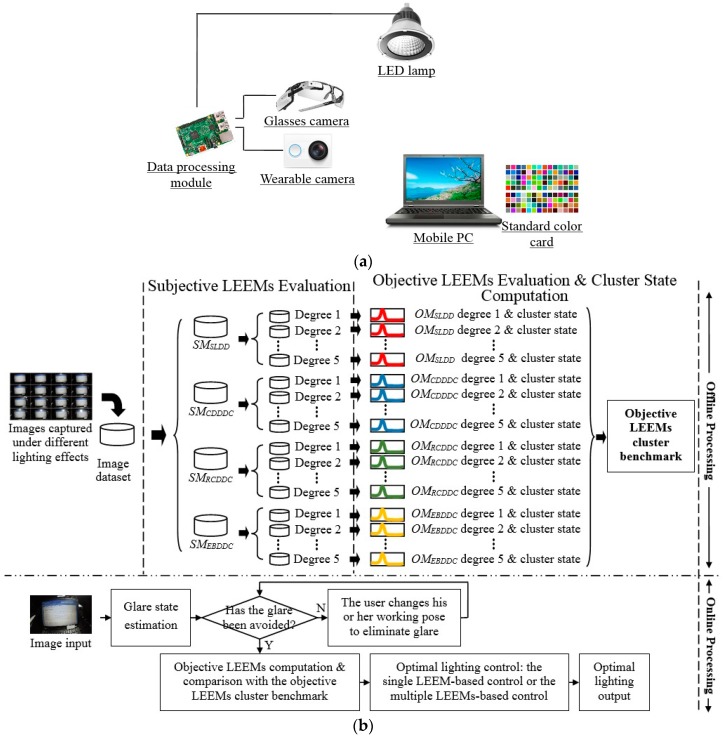
The design of proposed intelligent LED lighting system: (**a**) The hardware design of the intelligent lighting system; (**b**) The information processing flow chart of the proposed system.

Figure 3a shows the hardware design of the intelligent lighting system. A wearable visible light camera (or a glass camera) and its imaging data processing circuit are utilized by a user; a standard color card is pasted in the margin of the display; and a LED lamp is fixed near the user. When this system works, the camera is used to capture the image of display in front of the user. The data processing circuit [17] implements the image capture, the image analysis and the luminance control of the LED lamp. Then the LED lamp can carry out the optimal lighting output. Currently, the captured images can be transmitted to the data processing circuit by a wireless network [18]; while the connection method between the data processing circuit and the LED lamp is a cabled network. The color card is used to provide the color evaluation benchmark for the imaging sensor; and the basic color selection of it is decided by the typical desk operation task. The visual flicker of display should be removed by the proper setting of display output frequency. It is supposed the luminance output of display is stable when the desk operation task is carried out.

Figure 3b shows the working flow chart of the proposed system. The system works under an offline processing mode and an online processing mode. When implementing the offline processing mode, first a lighting effect image dataset is built. This dataset traverses most of the typical imaging lighting effects of the desk application. Second, a series of subjective evaluations are performed according to different subjective LEEMs to classify the dataset above into several sub-sorts. For example, four subjective LEEMs are assessed and the classification degree of each LEEM is five; thus 4 × 5 sub-datasets can be obtained. Third, the objective LEEMs are computed for the datasets above and their cluster centers are also analyzed. Then the cluster benchmarks of the objective LEEMs can be obtained.

When implementing the online processing mode, first the imaging sensor is used to capture the lighting effect image. Then the glare is eliminated from the proposed application. After that the objective LEEMs are computed and their calculation results are compared with the objective LEEMs cluster benchmarks. Finally, the optimal lighting control, i.e., a single LEEM-based or a multiple LEEMs-based control, can be used to get a kind of optimal lighting effect output.

## 3. The Key Techniques of Intelligent Lighting System

### 3.1. The Subjective Lighting Effect Evaluation Method

The research target of the subjective lighting effect evaluation experiment is to accumulate the image sub-datasets of the subjective LEEMs with different evaluation degrees. Then these sub-datasets can be regarded as the computation benchmarks for the following objective LEEMs computation. To achieve that target, first the typical lighting effect image sub-datasets are built; then the subjects are asked to implement the subjective lighting effect evaluations.

#### 3.1.1. The Typical Lighting Effect Image Dataset

A typical lighting effect image dataset is built. This image dataset is captured by a wearable camera in a dark room. The wall of the dark room is pasted by some light wave absorbing materials thus the lighting environment can be assessed and controlled elaborately. The application prototype of lighting system is shown in Figure 2. Without loss of generality, let us take the display read and monitoring task of desk application as an example. When the subject is performing the related tasks, different lighting conditions will be provided by a LED lamp constantly. Here “different lighting conditions” mean various kinds of settings of the LED output intensity or direction. The output intensity can be tuned by the control circuit. The output direction of LED lamp is also changeable because the user can move his or her working position. For example a mobile PC can be moved by user. To guarantee the application popularity, we do not restrict the lighting intensity output and the user’s working pose. The subjects can change these factors by their habits.

#### 3.1.2. The Subjective Lighting Effect Evaluation Experiment

**Figure 4:**
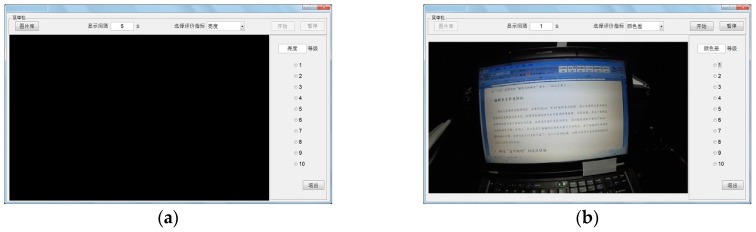
The interface photos of the subjective lighting effect evaluation experiment software: (**a**) The initial software interface; (**b**) The interface of the subjective lighting effect evaluation experiment.

The subjective lighting effect evaluation experiment can be carried out during the collection course of the typical lighting effect image dataset. An experimental software is developed to guide that experiment course and record the subjective evaluation results. Figure 4 shows two software interface photos. In Figure 4, (a) is the initial interface of the subjective evaluation experiment software; (b) is the interface of the subjective lighting effect evaluation experiment. This software was developed by Dr. Heng Zhang in Astronaut Research and Training Center of China in 2008. It was written by C++ and Matlab. The basic functions of this software include: first it can record and play the image dataset which is captured by a camera. Second, this software can select subjective evaluation index and set the evaluation reaction time of subject. Third the subjects can use it to score with respect to the subjective LEEMs. The largest score is 10. The subjective evaluation results can be saved in the local path of experiment computer by a file.

When performing the subjective evaluation experiment four LEEMs are considered. They include *SM_SLDD_*, *SM_CDDDC_*, *SM_RCDDC_*, and *SM_EBDDC_*. In this paper, the evaluation degree of each index is 5: degree 1 means the worst evaluation effect; while degree 5 means the best evaluation effect. For example, regarding the index *SM_EBDDC_*, the evaluation degree 5 means the blur degree of the display content almost cannot be noticed; differently, the evaluation degree 1 means the blur degree cannot be tolerated. From Figure 3b it can be seen after the subjective evaluation experiment, 4 × 5 image sub-datasets can be built. These datasets can be used to implement the objective LEEMs computation in the following processing steps. The glare is intolerant in our proposed application. That means once the glare is detected the system will notify the user to change his or her working pose to eliminate this glare. Thus it is not necessary to evaluate the subjective glare degree in this paper.

### 3.2. The Objective Lighting Effect Evaluation Method

Five objective LEEMs are computed to realize the lighting effect perception. Among these LEEMs, the indices *OM_SLDD_*, *OM_CDDDC_*, *OM_RCDDC_*, and *OM_EBDDC_* are used to compute the lighting effect; while the index *OM_GDD_* is employed to identify glare.

#### 3.2.1. The Surface Luminance Degree of Display

Like an imaging luminance meter, the visible light camera can be used to evaluate the surface luminance degree of display after luminance calibration. The International Commission on Illumination has defined two colorimetry coordinates: the 1931 CIE-RGB coordinate and the 1931 CIE-XYZ coordinate. The image luminance can be obtained by the 1931 CIE-XYZ coordinate directly. In the 1931 CIE-XYZ coordinate, the *Y* component stands for the luminance of color; it can be estimated by the relationship: *Y* = −1.7392*R* + 2.7671*G* − 0.0279*B*, where *R*, *G*, and *B* are the color components in the 1931 CIE-RGB coordinate. However, the commercial camera always has its own RGB spectrum response curve which is different from the standard response curves of two color coordinates mentioned above. In this paper the color coordinate of the commercial camera is called the imaging RGB color coordinate. Thus it is necessary to design a method to estimate the transformation rule between the 1931 CIE-XYZ coordinate and the imaging RGB color coordinate.

To realize the luminance measurement with a camera, a color calibration method is designed. A standard Munsell color card is placed near a standard lamp (its output power is 500 W and its spectrum scope is about 350 nm~800 nm), and a camera is used to shoot that color card. Then the color card can provide a color benchmark for the color measurement [19]. Table 1 shows part of the color components of the color card which uses the 1931 CIE-XYZ color coordinate. These color components can be measured by a spectrophotometer or provided by the color card manufacturer directly. More than 20 color cards are used in this experiment. The spatial distance between the color card and the standard lamp is about 1.0 m. All these experiments are carried out in a dark room. For each measurement result, the transform relationship between the imaging RGB color coordinate and the 1931 CIE-XYZ coordinate can be defined by (1) or (2). The least squares method [20] in (3) can be used to compute *M*. Finally, for any targets, the image luminance can be estimated by *L*. Here *L* is an estimation of the *Y* component in the 1931 CIE-XYZ coordinate:

(1) $$\left[\begin{array}{c} L_{1}\\ L_{2}\\ \ldots \\ L_{n} \end{array}\right]=\left[\begin{array}{ccc} R_{1} & G_{1} & B_{1}\\ R_{2} & G_{2} & B_{2}\\ \ldots & \ldots & \ldots \\ R_{n} & G_{n} & B_{n} \end{array}\right]\left[\begin{array}{c} m_{1}\\ m_{2}\\ m_{3} \end{array}\right]$$

(2) L=X×M

(3) $$M={\left(X^{T}X\right)}^{-1}X^{T}L$$

where *L* = [*L*_1_ *L*_2_ … *L_n_*] is the object luminance vector, it also stands for the *Y* component in the 1931 CIE-XYZ coordinate; [*R_i_ G_i_ B_i_*] (*i* = 1, 2, …, *n*) is the coordinate in the imaging RGB color coordinate; *X* is a color matrix, here *X* = [*R G B*]; to increase the computational precision, *X* also can be set by [*1 R G B RG RB GB R*^2^ *G*^2^ *B*^2^ *RGB*]; here *R*, *G*, and *B* are the color components in the imaging RGB color coordinate; *M* is an unknown matrix.

When calibrating the luminance of a camera, a luminance meter is utilized. In this paper the distance between the luminance meter and the target is also about 1.0 m. The relationship between the image luminance and the target luminance can be written using Equation (4), which can also be rewritten using Equations (5) and (6). If the target luminance *D* is measured by the luminance meter and the image luminance *L* can be calculated by Equation (2), in addition the variables *F* and *T* are known; then the variables *v* and *w* can be estimated easily: both the camera and the luminance meter are used to observe the same target, then a series of measurement results of the image luminance *L* and the target luminance *D* can be obtained; finally the relationship between the variables *v* and *w* can be fitted by the least squares method again. In this paper the observed target is the standard Munsell color card. Finally, the target luminance degree can be estimated by Equation (7):

(4) $$D=v\mathrm{lg}\left(\pi \tau TL/4F^{2}\right)+m$$

(5) $$D=v\mathrm{lg}\left(TL/F^{2}\right)+w$$

(6) w=vlg(πτ/4)+m

(7) $$OM_{SLDD}=D$$

where *ν* is the contrast ratio; *D* is the target luminance; *L* is the image luminance; *τ* is the optical transmittance of camera lens; *T* is the camera exposure time; *F* is the camera light ring coefficient, *F* = *f*/*d*; *f* is the camera focus, *d* is the size of light aperture; *m* is a coefficient.

#### 3.2.2. The Color Deviation Degree of Display Content

Because the object will absorb and reflect the spectrum components selectively, different objects will have different color characters under the same light source; similarly, as the light source has different spectrum components, the same object will also present different colors under different light sources. To assess the luminance effect, it is necessary to evaluate the color deviation degree of display. A Standard White Color Card (SWCC) is pasted in the margin of display. After a primary evaluation, in this paper it can be thought the SWCC can be identified by the visible light camera easily under a complex environmental light due to its excellent color contrast to the display margin. To identify the color deviation degree between the SWCC image block and the standard white color, first the camera searches and captures the imaging content of SWCC; then the color of SWCC block is transformed from the imaging RGB color space to the L*a*b* color space [21]; finally the Euclidean distance between the SWCC image block and the standard white color in (8) is used to assess the color deviation degree. The L*a*b* color space is utilized here because of its good color uniformity:

(8) $$OM_{CDDDC}=\sqrt{{\left(L_{1}-L_{2}\right)}^{2}+{\left(a_{1}-a_{2}\right)}^{2}+{\left(b_{1}-b_{2}\right)}^{2}}$$

where *L*_1_, *a*_1_, and *b*_1_ are the components of the SWCC image block in the L*a*b* color space; *L*_2_, *a*_2_, and *b*_2_ are the components of the standard white color in the L*a*b* space.

#### 3.2.3. The Region Contrast Degree of Display Content

The contrast reflects the salience and the difference degree between the image pixels of foreground and background. To improve the robustness of contrast evaluation, both the color region contrast [12] and the gray region contrast [22] are considered. Equation (9) shows the computation method of the gray region contrast. That contrast is a kind of Michelson contrast [23] which can measure the metric of periodic pattern. When estimating the gray region contrast, many points are sampled stochastically from the original image; then Equation (9) can be used to estimate the gray region contrast. Equation (10) shows the calculation method of the color region contrast. This function measures the root mean enhancement contrast in the log domain. It reflects the difference between the center pixel and its neighbors. The entire pixels in image will be used to compute that metric. Finally, the region contrast degree of display content can be defined by the linear combination of the gray region contrast and the color region contrast in (11):

(9) $$OM_{GC}=\frac{1}{N}\sum_{k=1}^{N}\left[\left(I_{k}^{\max}-I_{k}^{\min}\right)/\left(I_{k}^{\max}+I_{k}^{\min}\right)\right]$$

(10) $$OM_{CC}=\frac{\beta }{k_{1}k_{2}}\sqrt{\sum_{i=1}^{k_{1}}\sum_{j=1}^{k_{2}}{\left[\frac{\mathrm{lg}\left|I_{i,j}-\sum_{c=1}^{3}\lambda_{c}\left(I_{c1}+I_{c2}+\ldots +I_{cn}\right)/n\right|}{\mathrm{lg}\left|I_{i,j}+\sum_{c=1}^{3}\lambda_{c}\left(I_{c1}+I_{c2}+\ldots +I_{cn}\right)/n\right|}\right]}^{\alpha }}$$

(11) $$OM_{RCDDC}=w_{1}\times OM_{GC}+w_{2}\times OM_{CC}$$

where *I_k_^max^* and *I_k_^min^* are the maximum and the minimum gray values of the *k^th^* image block; *k*_1_ × *k*_2_ is the image block numbers of whole image, *I_i,j_* is the center image gray intensity; *n* is the pixel number of image block, (*I_c_*_1_ + *I_c_*_2_ + …+ *I_cn_*)/*n* is the average intensity of image block, *λ_c_* denotes the weights for each color component, here *λ_c_* can be *λ_r_*, *λ_g_*, *λ_b_*, and *λ_r_* = 0.299, *λ_g_* = 0.587, *λ_b_* = 0.144; *α* and *β* are parameters, *α* = 0.8 and *β* = 1000; *w*_1_ and *w*_2_ are weights, *w*_1_ = 0.2, and *w*_2_ = 0.8.

#### 3.2.4. The Edge Blur Degree of Display Content

The edge blur degree represents the definition of image edges. As for the proposed application, the edge blur comes from the weak eyesight of subjects, the low luminance of the display, or even disturbances of the non-uniform environment lighting output. To evaluate the edge blur degree, the computation method in [14] is utilized. The computation equation of edge blur is shown in (12). This metric evaluates the edge blur by computing the edge spread degree of the edge points. When designing this lighting system, the moiré pattern [24] may happen. The moiré pattern is generated from the micro-grooved structure of imaging sensor. It will occur when two or more images are combined nonlinearly to create a new superposition image with a magnified period in comparison with the original images. Thus the moiré pattern should be identified and distinguished from the image blur phenomenon. Regarding this proposed application, the moiré pattern can be identified by the image filtering technique and decreased by the image interpolation method [25]:

(12) $$OM_{EBDDC}=\underset{i\in \Theta }{\max}\left\{\arctan\frac{\left[I\left(x_{i1},y_{i1}\right)-I\left(x_{i2},y_{i2}\right)\right]}{w_{i}}\right\}$$

where Θ is the set of image block, *I*(*x_i_*, *y_i_*) is the gray value of the *i*^th^ image block in position (*x*, *y*), *w_i_* is the width between the edge-spread points (*x_i_*_1_, *y_i_*_1_) and (*x_i_*_2_, *y_i_*_2_).

#### 3.2.5. The Glare Degree of Display

A glare may occur in the screen when the display or the LED lamp is moved by the user. The glare may seriously affect the visual output effect. For example, the display contents, such as the characters or the content details, may be sheltered by the glare region; in addition, the glare will also evoke the visual fatigue or the bad emotion of user; therefore it is necessary to avoid it. In general, the glare can be classified into two types [15], i.e., the disability glare and the discomfort glare. As for the proposed desk application, only the discomfort glare is considered. Many indices [26] have been invented to assess the degree of manmade glare, including the British Glare Index (BGI), the CIE Glare Index (CGI), and the Unified Glare Rating (URG) etc. In this paper, only the existence or the nonexistence of glare is considered. If a glare is identified by the system, an alarm will be reported to the user; and then the user should change his or her working pose to avoid it.

To detect and evaluate the glare degree, first the fixed threshold-based image segmentation method is utilized to search and mark the glare region. The computation equation is shown in (13). The threshold can be gotten from the practical experiment test of typical desk application. The initial binary segmentation image can be gotten by this method. Then the mathematical morphology can be employed to expand the region and the edge of the segmented binary image. In this paper the segmented high-lighted glare regions can be defined as the foreground region; while other regions can be defined as the background region. Finally, the pixel numbers inside of the foreground region will be counted. Equation (14) shows the identification method of the glare region: if the pixel ratio between the number of foreground pixel and that of the total pixel is large than a threshold, the foreground can be regarded as the glare region:

(13) $$I_{S}=\{\begin{array}{cc} 0 & I>T_{S}\\ 255 & else \end{array}$$

(14) $$OM_{GDD}=\{\begin{array}{cc} 1 & C(I_{S})/C(I)>T_{G}\\ 0 & else \end{array}$$

where *I* is the image grey intensity of the original image, *T_S_* is a threshold, *I_S_* is the binary segmentation result of the original image, function *C*(*) computes the pixel number of image block “*”, *T_G_* is a threshold, here *T_G_* = 0.08.

### 3.3. The Intelligent Lighting Control Methods

**Figure 5:**
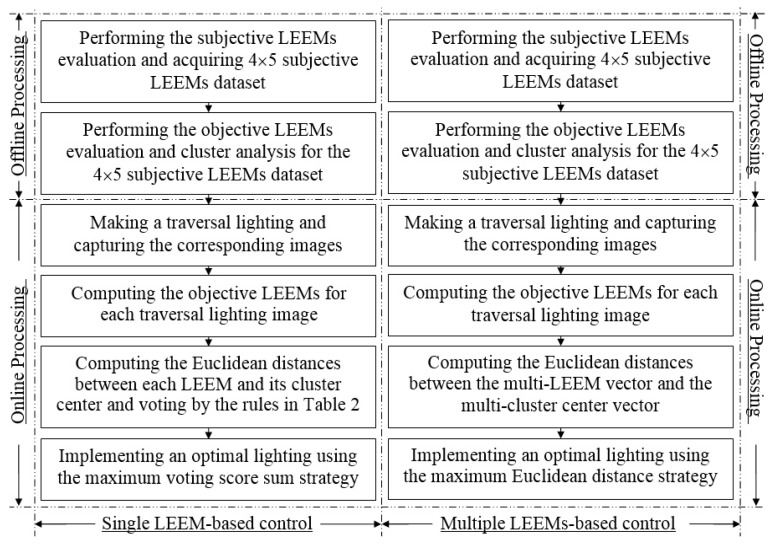
The proposed flow chart of the intelligent luminance control methods.

After the quantitative evaluation of the lighting environment, a luminance tuning of LED lamp should be made. Currently only the luminance intensity of LED lamp is tuned. The control rules of the lighting system are: first, the glare which appears in the display should be detected and avoided; second, a kind of optimal luminance control will be implemented. Here the optimal control means a lighting effect without over-illumination or over-darkness. Figure 5 shows the design flow chart of the intelligent luminance control methods. Both a single LEEM-based control and a multiple LEEMs-based control are developed. Here, the optimal luminance is only a relative optimization. When searching the optimal luminance, because no priori of the environment luminance is provided, this system will make a traversal luminance control firstly. For example, the system will implement ten kinds of different luminance controls of LED lamp and record all the images captured under these luminance conditions. Here the LED output of different intensities can be controlled by the corresponding current or voltage. Then the system will compute and evaluate all the LEEMs for each captured image and select only one optimal luminance result as the final control method.

The single LEEM-based method controls the lighting by assessing the single LEEM one by one. After the subjective evaluation of the typical lighting effect image dataset, 4 × 5 image sub-datasets can be accumulated. The objective LEEMs are used to compute the objective evaluation results for each image sub-dataset above; then the corresponding benchmark cluster centers of them can be gotten. The cluster centers can be estimated by the K-means method [27]. When implementing the lighting control, the Euclidean distance [28] and the voting rule are utilized: four objective LEEMs are computed for each image captured from the traversal lighting effects; then the Euclidean distance between each new computed objective LEEM and the corresponding benchmark cluster center is computed; if the Euclidean distance is located in a typical threshold scope, a quantitative score will be assigned. The quantitative method is shown in Table 2. Finally, the optimal lighting control will be that one which has the maximum score sum of four LEEMs.

The multiple LEEMs-based method controls the lighting by the assessment of the multiple LEEMs together. Regarding this method, a high-dimension evaluation vector can be build. For example, this vector can be written as [*OM_SLDD_ OM_CDDDC_ OM_RCDDC_ OM_EBDDC_*]. The K-means cluster method is used to find its high-dimension benchmark cluster center for the typical lighting effect image sub-datasets. When carrying out the lighting control, the images with the traversal luminance are captured and their corresponding high-dimension LEEMs vectors can be computed as the candidate control methods; then the Euclidean distance in (15) will be used to compute the distance between the benchmark cluster center and the corresponding LEEMs vector candidate. Then the optimal control will be that one which has the minimum Euclidean distance offset:

(15) $$\Delta D=\sqrt{\sum_{k=1}^{4}{\left(LF_{k}^{C}-LF_{k}^{O}\right)}^{2}}$$

where *LF_k_^C^* is the *k*th component of cluster center of the high-dimension vector; while *LF_k_^O^* is the *k*th component of the observation data of the high-dimension vector.

## 4. Experiments and Discussion

### 4.1. Experiments & Evaluations

#### 4.1.1. Experimental System and Experimental Environment

**Figure 6:**
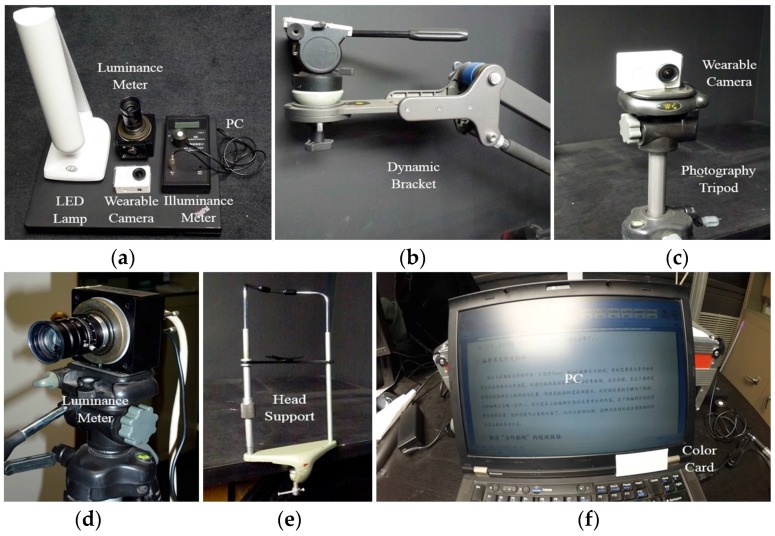
The photos of experiment apparatuses: (**a**) The basic experiment apparatuses; (**b**) The dynamic bracket; (**c**) The wearable camera and the photography tripod; (**d**) The luminance meter; (**e**) The head support; (**f**) The experiment computer and the standard white color card.

To evaluate the performance of proposed technique an experimental system was built. This experimental system works in a dark room. Figure 6 shows photos of this experimental system: a computer, a white light LED lamp, a luminance meter, an illuminance meter, and a CMOS camera are shown in (a); a dynamic bracket which is used to control the spatial position of camera is given in (b); a photography tripod with a camera is shown in (c); a luminance meter is shown in (d); a head support which is used to estimate the height of user’s head is given in (e); a PC and a SWCC (see Section 3.2.2) are shown in (f). When implementing the experiments, because the imaging performance of the glass camera is poor, a motion camera which has a better imaging sensor is utilized. The motion camera can be installed in the dynamic bracket or the photography tripod to imitate the observation state of human eyes. It is placed in front of a computer, and the LED lamp is used to provide the lighting source. This experiment system can be used to carry out the subjective lighting effect evaluation experiment and build the corresponding image datasets; it also can be employed to implement other human-involved experiments to assess the lighting tuning effect of proposed system.

**Figure 7:**
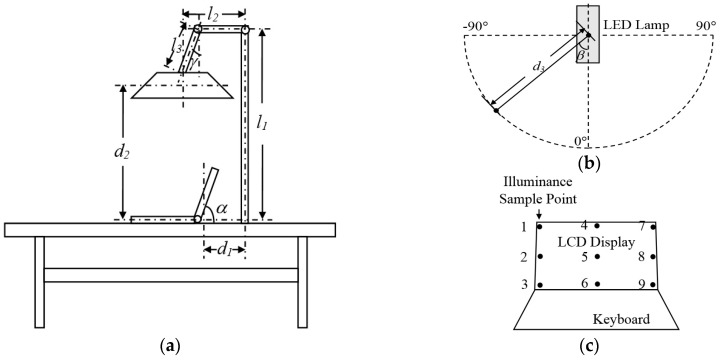

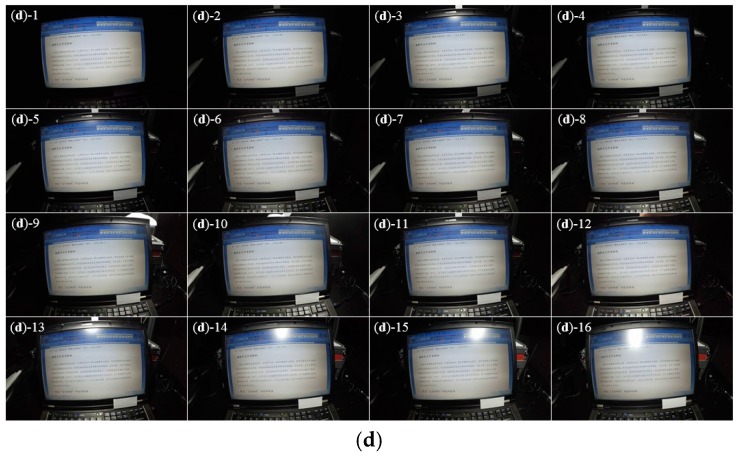
The experiment system sketch maps and its captured photos in different lighting conditions: (**a**–**c**) The sketch maps of the experiment system, where α ≈ 80°, *d*_1_ ≈ 20 cm, *d*_2_ ≈ 80 cm, *l*_1_ ≈ 90 cm, *l*_2_ ≈ 15 cm, *l*_3_ ≈ 1.5 cm, γ ≈ 5°; (**d**) The captured photo samples using the proposed experiment system.

**Figure 8:**
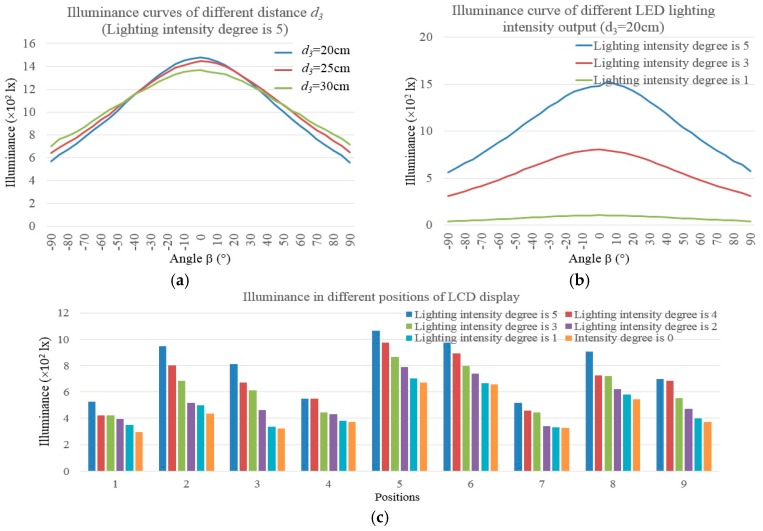
The illuminance distribution curves of LED lamp under different lighting outputs and measurement conditions: (**a**) The illuminance curves of different sample distances; (**b**) The illuminance curves of different LED lighting intensities; (**c**) The illuminance results in different sampling positions of the computer LCD display.

Figure 7 shows the sketch maps of the lighting experiment system and its captured image samples: Figure 7a,b are the sketch map of the lighting system, the black points in Figure 7b show the sample position of the illuminance meter. The sample scopes are from −90° to +90° and the sample interval is 5°; thus 37 sample points can be gotten. Figure 7c is the front view of the experiment computer; the black points mark the sample positions. Figure 7d shows the captured image samples and Table 3 gives examples of their lighting effect. In Table 3, the average illuminance means the average lighting intensity of the sample points; the illuminance uniformity means the intensity variance. The illumination scopes of LED lamp are from 1.0 lx to 40,000.0 lx; its color temperature ranges are from 3500 K to 6000 K. Figure 8 shows the illuminance distribution curves of the lighting system of Figure 7a. Without loss of generality, the illuminance degrees of LED lamp are supposed to have 5 degrees: degree 5 has the strongest output effect while degree 1 has the weakest output effect. The sample distances *d*_3_ in Figure 7b are: degree 1 is 20 cm, degree 2 is 25 cm, and degree 3 is 30 cm.

#### 4.1.2. Subjects

Eight males (ages from 25 to 35; heights from 165 cm to 175 cm; weights from 60 kg to 75 kg) were selected to participate in the intelligent lighting effect evaluation experiments. After a primary ocular examination, none of the subjects had any ophthalmopathies, such as the myopia, hyperopia, or color blindness, etc. Their uncorrected eyesight should be better than 0.8. Before the subjects participated in any human-involved experiments, the entire experiment procedures were described to them clearly; and after authorization by the Ethics Committee of the School of Biological Science and Medical Engineering, Beihang University, the experiments could be carried out.

#### 4.1.3. Realization Results of the Intelligent Lighting System

The human-involving experiments are used to create the subjective lighting effect evaluation dataset. The subjects are asked to evaluate the lighting effect according to their subjective cognition. Currently, the evaluation experiments only use the single factor evaluation method to accumulate the corresponding dataset. Here the single factor experiment asks the subjects to evaluate the single LEEM one by one for each lighting scene. The subjective evaluation degrees are divided into five levels: very good, good, fair, bad, and worst. Their corresponding quantification degrees are 5, 4, 3, 2, and 1. Each subjective LEEM has its own subjective evaluation criteria. For example, regarding *SM_SLDD_* and *SM_RCDDC_*, the larger their subjective sense diversities are, the larger their degrees should be; and as for *SM_CDDDC_* and *SM_EBDDC_*, the smaller their subjective sense diversities are, the larger their scores should be. After the subjective lighting effect evaluation, twenty image sub-datasets can be built. For example, Figure 7d shows the image dataset samples and Table 4 shows the single LEEM subjective evaluation results of Figure 7d((d)-5–(d)-8).

**Figure 9:**
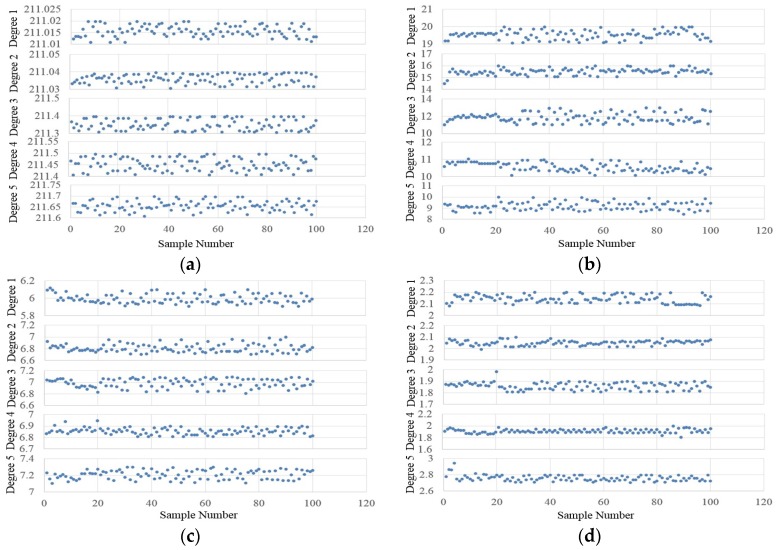
The computation results of the objective LEEMs: (**a**) The objective evaluation results of *OM_SLDD_*; (**b**) The objective evaluation results of *OM_CDDDC_*; (**c**) The objective evaluation results of *OM_RCDDC_*; (**d**) The objective evaluation results of *OM_EBDDC_*.

After the subjective evaluation image sub-datasets have been built, the objective LEEMs can be computed. Equations (16) and (17) show the estimation results of the camera luminance calibration, where *F* = 3.6, *T* = 2.083 ms. Figure 9 shows the computation results of the objective LEEMs: (a–d) are the results of *OM_SLDD_*, *OM_CDDDC_*, *OM_RCDDC_*, and *OM_EBDDC_* in different subjective evaluation degrees, respectively. The K-means method is used to calculate the cluster centers of each objective LEEM under different lighting effect degrees. Table 5 shows the statistic results of these data above. Their means and variances are computed here. When carrying out the optimal lighting control, the voting rule and the quantitation method in Table 2 are used. For example, regarding two images, the objective quantitative scores of the first image are: *OM_SLDD_* = 4, *OM_CDDDC_* = 4, *OM_RCDDC_* = 3, and *OM_EBDDC_* = 4; the quantitative scores of second image are: *OM_SLDD_* = 2, *OM_CDDDC_* = 1, *OM_RCDDC_* = 2, and *OM_EBDDC_* = 3. Thus the first image has a better lighting effect because of its better voting result: the voting score sums of two images are 4 + 4 + 3 + 4 = 15 and 2 + 1 + 2 + 3 = 8, respectively:

(16) $$M={\left[\begin{array}{ccc} 0.0687 & 0.2377 & -0.0301 \end{array}\right]}^{T}$$

(17) $$D=30.537\times \mathrm{lg}\left(TL/F^{2}\right)+66.4003$$

The high-dimension data samples and their cluster centers of the multiple LEEMs are shown in Table 6. The multiple LEEMs-based control assesses the lighting effect by considering all the objective LEEMs together. In Table 6, three sample data are illustrated for each subjective evaluation degree. The high dimension K-means method is used to compute the benchmark cluster centers. When a new image is captured, first its LEEMs vector is calculated. Then the Euclidean distance between that vector and the corresponding benchmark cluster center will be used to find the optimal lighting control.

For example, if the subjective lighting evaluation degree 5 is defined as the optimal lighting effect; regarding two images, the first one gets its LEEMs vector as [211.0197 19.6238 5.9761 2.1751]; while the second one calculates its vector as [211.6442 9.1758 7.2765 2.7980]; and the objective LEEMs benchmark cluster center of the subjective evaluation degree 5 is [211.6554 9.1785 7.2084 2.7585], thus the second image has a better lighting effect because of its smaller Euclidean distance offset.

**Figure 10:**
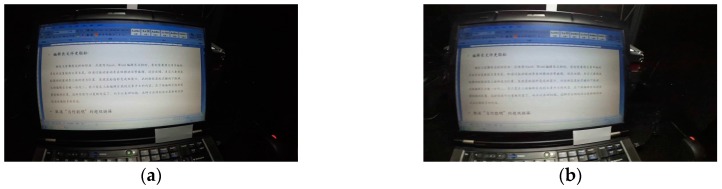
The lighting effect outputs before and after the single LEEM-based control: (**a**) The lighting effect output before the application of the single LEEM-based control; (**b**) The lighting effect output after the application of the single LEEM-based control.

Because it is difficult to completely recover the light field from one image, a kind of “local” optimal control is used here: ten kinds of typical traversal lighting controls are implemented to get a series of lighting images. Then the single LEEM-based or the multiple LEEMs-based control is used to select only one control method which has the minimum distance offset between the candidate and the predefined optimal lighting benchmark as the final control. The traversal control of LED is to increase or decrease its luminance output little by little. Figure 10 shows a control result of the proposed system: (a) shows the original lighting effect; (b) is the photo after the implementation of the single LEEM-based control. Obviously, (b) has a better lighting output. Table 7 shows the LEEMs comparison results before and after the lighting control of Figure 10a. Obviously its lighting effect can be improved according to Table 6. The control technique of the multiple LEEMs-based method can get a similar processing result.

#### 4.1.4. Processing Results of Glare

**Figure 11:**
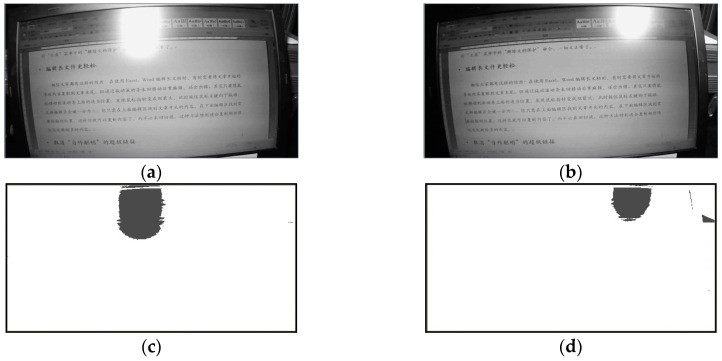
The glare image samples and their segmentation results: (**a**,**b**) The glare cases of improper lighting; (**c**,**d**) The binary segmentation results of (**a**,**b**), respectively.

The glare should be avoided for any desk applications because of its negative influences on the visual operation task. Figure 11 shows two glare images and their processing results by the proposed system. In Figure 11, it can be seen the image details are sheltered by the high-lighted regions. If the glare region is large than a threshold an abnormality should be reported; then the user will change his working pose or just move the spatial position of the computer to eliminate the glare. From Figure 11, the glare can be identified if the gray segmentation threshold is set from 210 to 230. Regarding a desk application, the glare mainly comes from the LED source reflection of the screen or other interferential light sources of the environment. Obviously, the automotive recognition of glare has the practical engineering application meaning for the design of intelligent lighting system.

#### 4.1.5. System Application Evaluations

To evaluate the performance of the proposed intelligent lighting system, a human-involved experiment is performed. First, a subjective evaluation experiment of the static lighting is performed. Here the static lighting means the traditional lighting method, i.e., no intelligent lighting control is executed here. Its experiment procedures are: the subjects are asked to stay in front of a display in the dark room; the LED lamp provides a static lighting environment. In that situation the subjects are asked to read the display content (see Figure 7d) and give their subjective assessments of different lighting effects. No intelligent lighting tuning is made during that process. Second, the proposed intelligent lighting system is used to improve the lighting effect. The single LEEM-based control is carried out here. Once the intelligent system stops work, the subjects are required to give their subjective evaluations of the corresponding lighting effects again. Then a comparison of the lighting effect degree before and after the application of the proposed intelligent lighting control can be made. During the experiment procedure above, each display-reading task will last 120 s and the reading content is the modern Chinese article; then the subjects will perform the subjective evaluation mission.

Table 8 shows the comparison results of the lighting effect evaluation experiment. From the table it can be found that each index of the subjective evaluation factor can be improved apparently by using the proposed intelligent lighting control technique. The multiple LEEMs-based control method can get the similar experiment results.

In this paper, another Chinese article-reading task is used to evaluate the performance of proposed system. In this experiment the visual fatigue degree and the reading error rate [29] are used as the evaluation indices. The visual fatigue degree represents the subjective ocular weariness feeling of subject; in physiology, it behaves as the eye itch, the eye pain, the eye dry, the tearing, the photophobia, or the ocular foreign body sensation, etc. A 5-degree score method is used, where a 5 means the visual fatigue is serious and a 1 means it is slight. The reading error rate reflects the visual cognition ability of subject. Its value will increase as the visual function falls. It can be computed by using the number of improperly read characters divided by the total number of characters in an article. The experiment was as follows: a subject is asked to read a Chinese article twice. The subject does not know any information about this article before the experiment. Each experiment lasts 10 min. The reading speed should be > 300 Chinese characters/minute. An old Chinese article is used so that the phrases will not be familiar to the subject. As a result, the subject has to observe every Chinese character carefully before he reads it out. When this experiment is carried out for the first time, the lighting state is static, whereas the second time the intelligent lighting control is applied. The experiment organizer will record the corresponding evaluation results during the experiment course. Table 9 shows the experiment results. From Table 9, it can be seen the proposed lighting system can effectively decrease both the visual fatigue and the reading error rate.

### 4.2. Discussion

The lighting system plays an important role in practical applications, especially for the case of the hermetic cockpit design or narrow working space applications. In our past research work [7], an ergonomics experiment was designed and implemented to assess the importance of the lighting factor for the desk operation of a hermetic aerospace cockpit. In that experiment, among those factors, including the observation distance, the observation angle, the Chinese character size, the Chinese character color or its background color, and the lighting degree, the lighting degree played the second importance role in that application. Recently, many other research works [30] have also disclosed the distinct relationship between ocular diseases and improper lighting effects. Some ocular diseases such as myopia, glaucoma, or abnormal intraocular pressure, etc. can all be aggravated by improper lighting. Besides, even when the lighting effect is proper, the different working habits of subjects also need some typical lighting output settings. Thus the lighting has become a fatal factor for the evaluation and the design of desk system applications.

A subjective evaluation experiment is used to build the benchmark image datasets for the following objective evaluation computation. When the accumulation of benchmark datasets starts, the subject is asked to watch the display in a dark room; an article reading task is shown in the screen and the lighting effect will be tuned constantly. During that process the subject is requested to give his or her subjective evaluation scores of the lighting effect; a software system guides the experiment procedures and records the corresponding results. Here it is wrong to build that benchmark datasets by showing the subject the camera-captured image in screen and asking them to score the displayed images. Because the camera imaging mechanism is different from the visual cognition mechanism of human; the images captured by camera are only a kind of degraded processing result of the imaging sensor and the optics system. The subjective evaluation results are related with the subject’s eyesight, age, and training state, etc.; thus the proposed intelligent lighting system can also encapsulate and record the ocular habits and the physiological characteristics of system users.

Four objective LEEMs are employed to compute the lighting effect quantitatively: the display luminance, the image color difference, the image contrast, and the image blur. These metrics also correspond to the blind image quality evaluation metrics. That means they are the content-independent [31] image feature describers. The reason that these metrics are selected as the evaluation indices is two-fold. The first fact is the consideration of the practical application. In this paper, the desk experiment is a mesopic vision and photopic vision application case [32]. The luminance scope of mesopic vision is about 0.001 cd/m^2^ to 10.0 cd/m^2^; while the luminance scope of photopic vision should be larger than 10.0 cd/m^2^. At that luminance level, the image contrast and the image blur are fit for representing the imaging quality effectively. The second fact is that the luminance, the color difference, and the contrast also belong to the traditional ergonomic investigation indices used for ocular ability assessment [33]. Thus the display luminance, the image color difference, and the image contrast should also be selected as the evaluation indices to measure the lighting effect from the ergonomics research point of view.

**Figure 12:**
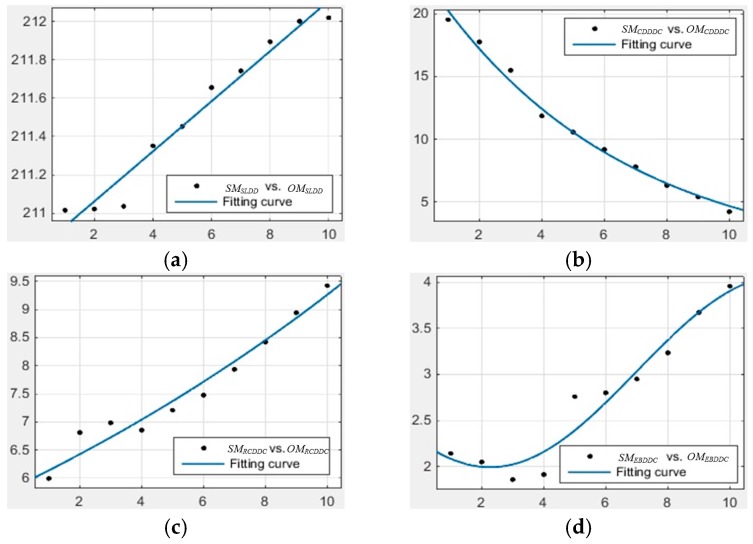
The fitting relationship curves between the subjective evaluation index and the cluster center of objective evaluation index: (**a**) The fitting result curve between *SM_SLDD_* and the cluster center of *OM_SLDD_*; (**b**) The fitting result curve between *SM_CDDDC_* and the cluster center of *OM_CDDDC_*; (**c**) The fitting result curve between *SM_RCDDC_* and the cluster center of *OM_RCDDC_*; (**d**) The fitting result curve between *SM_EBDDC_* and the cluster center of *OM_EBDDC_*.

In this paper, the number of subjects was relatively small because of our limited financial resources, thus we only call the related experiments “human-involved” experiments rather than ergonomics experiments. By performing these experiments some primary laws of vision lighting effect can be deduced. Currently, these results cannot be considered universal laws; however they still can illuminate the potential scientific application value of our proposed method and system. Figure 12 shows the primary fitting relationship curves between the subjective evaluation index and the cluster center of the objective evaluation index.

The horizontal axis is the subjective evaluation degree of lighting effect; the vertical axis is the objective evaluation result of lighting effect. In Figure 12, (a) is the fitting curve between the index *SM_SLDD_* and the cluster center result of *OM_SLDD_*; (b) is the fitting curve between the index *SM_CDDDC_* and the cluster center result of *OM_CDDDC_*; (c) is the fitting curve between the index *SM_RCDDC_* and the cluster center result of *OM_RCDDC_*; and (d) is the fitting curve between the index *SM_EBDDC_* and the cluster center result of *OM_EBDDC_*. The exponential functions are used to fit the results in (a–c), while a Fourier series are employed to fit the result in (d). By using the curves below, the cluster centers of objective indices can be forecast to implement some intelligent lighting system controls in the future.

The glare index is utilized in this paper to find and eliminate the glare-contaminated image blocks from the accumulated image datasets. In the proposed experiments, the glare mainly comes from the screen reflection of the strong lighting source. The improper spatial positions of display and light source will create such kind of phenomena. However, other cases, for example outdoor lightning, external strong lights, or a lamp flash which appears near the user’s visual field will also create glares for the system user. Currently, the *OM_GDD_* computation is only implemented within the display image area. In the future, the glare will be computed for the entire image scope captured by the wearable camera; thus other glare cases can be recorded and processed. In some application cases, if the glare cannot be avoided, the degree evaluation of it should be considered. A kind of degree evaluation standard for glare is proposed by CIE 112-1994 [34]. Thus in the future our system can perform quantitative evaluations of glare degree.

Two kinds of control methods are developed to implement the optimal control of lighting systems. After some experimental evaluations, the single LEEM-based control can approach the optimal lighting if the accumulated image dataset is small; differently, the multiple LEEMs-based control method is appropriate for realizing the optimal lighting control if the accumulated dataset is large. This phenomenon can be explained by the fact that the former method uses more man-made control parameters than the latter method. From the engineering application point of view, the use of the single LEEM-based method is obviously more complex than the use of the multiple LEEMs-based method; however, the small dataset requirement of the former method still makes it fit for the development of some low cost industrial lighting products. Other complex systems which demand elaborate lighting control can use the multiple LEEMs-based control. In future, the integrated control method which uses both the single LEEM-based and the multiple LEEMs-based control techniques can be developed.

**Figure 13:**
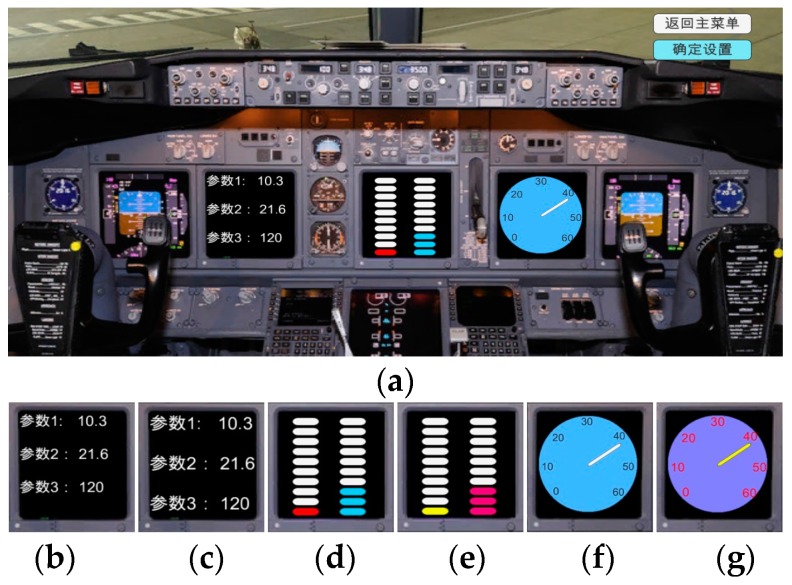
The airplane cockpit instrument panel application of the proposed system: (**a**) The simulation interface of instrument panel; (**b**,**c**) The parameter output interfaces which use different character sizes; (**d**,**e**) The progress bars which use different colors; (**f**,**g**) The circle dial plates which use different character sizes and background colors.

Figure 13 shows a simulation interface of an airplane cockpit instrument panel; the proposed lighting system can be used to assist the design of that panel. Figure 13a is the simulation interface; (b,c) are the parameter output interfaces which use different character sizes; (d,e) are the progress bars which use different colors; (f,g) are circular dials which use different character sizes and background colors. By using the proposed lighting system, it can be found the luminance intensity requirement of the combined images (b,d,f) is larger than the requirement of the combined images (c,e,g). This result may be explained by the fact that the image details of the former combination mode have a weaker imaging effect than that of the latter combination mode for human eyes. From this application it can be seen the proposed technique can be used for the industrial system design. With the predefined lighting degree, the tuning of LED lamp will always find ways to approach the optimal lighting effect. The LED lamp can be used as the main light source or only the lighting supplement device. And it can improve the task reliability.

**Figure 14:**
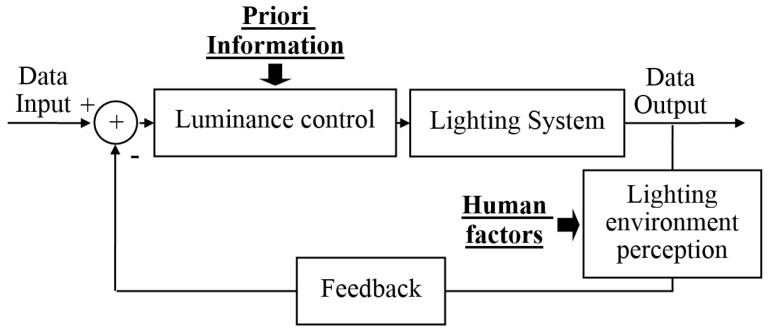
The schematic diagram of feedback control of the proposed intelligent lighting system.

To show the scientific background and the rationale of our proposed intelligent lighting system further, Figure 14 shows its feedback control schematic diagram. From Figure 3 and Figure 14 it can be seen that the proposed intelligent lighting control derives from the classic adaptive control model [35]. Our proposed control system has its own characteristics. First, it uses the blind image quality evaluation metrics to perceive the environmental lighting. A well designed blind image quality evaluation metric will be independent to the image content; i.e., it can only represent the essential attributes of an imaging scene. In contrast to other sensors, it also can get abundant detail information. Second, the human factors are encapsulated in this system by some mathematical tools. The human factors at least include the visual function of human eyes and the working habits of users; in many cases they even can reflect the user’s age, occupation, or gender. By the encapsulation of human factors, our proposed system can realize a real human-centric design. Third, the flexible luminance control methods are employed. Here the luminance control depends on the priori information. If the priori information is sufficient, for example the lighting environment and the working space are known (the corresponding information can be evaluated by the simultaneous localization and mapping technique [36] and the 3D reconstruction technique [37], etc.), then the 3D lighting design can be carried out by the precise computation. The global optimal lighting control can be realized in that situation. However, if the priori information are unknown or limited, our proposed local optimal control method can be utilized.

With the rapid development of wearable devices and intelligent product, the intelligent sensor has gradually changed the design concepts of objects used in our daily life. As an important branch, of this field, an intelligent lighting apparatus can realize friendly interactions with users. Intelligent lighting apparatus can have many applications. For example, some ocular diseases such as myopia or glaucoma can be alleviated by such a system; building lighting, medical lighting, and teleoperation lighting, etc., all need the application of the intelligent lighting control technique. As a result, the image analysis-based lighting evaluation and control technique definitely has good development prospects. In the past, the environmental perception abilities of intelligent sensors were limited. The traditional infrared sensor only can get one-dimensional perception information; differently, the imaging sensor can realize two-dimension environmental perception by using the blind image quality evaluation metrics. This innovative technique can expand the design concept of traditional intelligent lighting systems. In the future, the human-centric design will get great attention when developing this kind of system. For example, more image analysis techniques and ergonomics research results can be utilized to improve the system performance; other LEEMs such as the degree of noise contamination of images or the color temperature degree of LED lamps can also be applied, and the reaction time, the eye movement tracking state [38] or even the physiological signal feature [39] can also be studied to improve the integrated system output effect.

## 5. Conclusions

An intelligent lighting evaluation and control technique is proposed in this paper. Both a human-involved experimental method and the image analysis techniques are used to analyze the lighting effect. The subjective and the objective LEEMs are utilized to realize the optimal lighting effect evaluation: the image datasets of the subjective LEEMs are built; the distribution rules of the objective LEEMs are computed. Single LEEM-based and multiple LEEMs-based control techniques are also developed. The surface luminance degree of a display, the degree of color deviation of content, the region contrast degree of display content, and the edge blur degree of display content, are all used for the computation of the objective lighting effect. The glare can be identified automatically. By using the proposed system, the lighting effect of a desk application can be effectively improved.

## Figures and Tables

**Table 1:** The color component samples of the standard color card.

Color Coordinate	Cyan	Blue	Green	Red	Yellow	Orange	White
X	14.77	7.78	14.22	19.84	54.59	37.99	84.37
Y	19.55	6.16	22.88	12.41	58.45	30.44	89.25
Z	40.08	26.89	10.08	5.46	9.17	6.72	93.71

**Table 2:** The quantitation processing method of the single LEEM-based control technique.

Threshold Scope	[*T*_1_, *T*_2_]	[*T*_3_, *T*_4_]	[*T*_5_, *T*_6_]	[*T*_7_, *T*_8_]	[*T*_9_, *T*_10_] ^1^
Quantitative score	5	4	3	2	1

^1^ *C_i_* (*i* = 1, 2, …, 5) is the cluster center; *T_i_* (*i* = 1, 2, …, 10) is the threshold; and *T*_1_ < *C*_1_ < *T*_2_, *T*_3_ < *C*_2_ < *T*_4_, *T*_5_ < *C*_3_ < *T*_6_, *T*_7_ < *C*_4_ < *T*_8_, *T*_9_ < *C*_5_ < *T*_10_.

**Table 3:** The illuminance measurement results of Figure 7d((d)-5–(d)-8).

Image Name	Maximum Illuminance (×10^2^ lx)	Minimum Illuminance (×10^2^ lx)	Average Illuminance (×10^2^ lx)	Illuminance Uniformity (×10^2^ lx)
Figure 7d((d)-5)	6.03	1.23	4.94	2.47
Figure 7 d((d)-6)	7.01	1.54	5.73	2.42
Figure 7 d((d)-7)	12.74	4.91	9.38	3.62
Figure 7 d((d)-8)	14.75	5.16	11.71	5.30

**Table 4:** The single factor subjective lighting degree evaluation results of Figure 7d((d)-5–(d)-8).

Image Name	Single LEEM Subjective Evaluation Degree			
	*SM_SLDD_*	*SM_CDDDC_*	*SM_RCDDC_*	*SM_EBDDC_*
Figure 7d((d)-5)	2	2	3	3
Figure 7d((d)-6)	2	3	3	3
Figure 7d((d)-7)	3	3	4	4
Figure 7d((d)-8)	4	4	5	4

**Table 5:** The statistic results of the objective LEEMs in Figure 9.

**Metric**	***OM_SLDD_***					***OM_CDDDC_***				
**Degree**	**1**	**2**	**3**	**4**	**5**	**1**	**2**	**3**	**4**	**5**
Mean	211.0157	211.0360	211.3514	211.4528	211.6554	19.5305	15.4934	11.8511	10.5660	9.1785
Variance	6.0053 × 10^−6^	7.3102 × 10^−6^	1022.72 × 10^−6^	763.109 × 10^−6^	515.628 × 10^−6^	612.6363 × 10^−4^	709.9228 × 10^−4^	2959.6242 × 10^−4^	615.6026 × 10^−4^	1331.8018 × 10^−4^
**Metric**	***OM_RCDDC_***					***OM_EBDDC_***				
**Degree**	**1**	**2**	**3**	**4**	**5**	**1**	**2**	**3**	**4**	**5**
Mean	5.9951	6.8110	6.9854	6.8544	7.2084	2.1418	2.0492	1.8586	1.9126	2.7585
Variance	28.1307 × 10^−4^	49.3477 × 10^−4^	60.9873 × 10^−4^	8.5301 × 10^−4^	29.6785 × 10^−4^	12.1872 × 10^−4^	4.5413 × 10^−4^	10.3775 × 10^−4^	10.8874 × 10^−4^	14.3658 × 10^−4^

**Table 6:** The data samples and their cluster centers of the objective multiple LEEMs.

Subjective Lighting Degree	The Objective LEMMs Vector [*OM_SLDD_ OM_CDDDC_ OM_RCDDC_ OM_EBDDC_*]	The Cluster Center
1	[211.0122 19.4462 6.0243 2.1990]	[211.0157 19.5305 5.9951 2.1418]
1	[211.0133 19.6238 5.9806 2.1883]	
1	[211.0134 19.6238 5.9962 2.1404]	
2	[211.0333 15.2866 6.8128 2.0490]	[211.0360 15.4934 6.8110 2.0492]
2	[211.0342 15.2866 6.7730 2.0295]	
2	[211.0354 15.4643 6.7668 1.9936]	
3	[211.3667 12.0009 6.9177 1.8845]	[211.3514 11.8511 6.9854 1.8586]
3	[211.3323 11.9121 6.9079 1.8619]	
3	[211.3542 12.0009 6.9371 1.8672]	
4	[211.4667 10.8630 6.8696 1.9042]	[211.4528 10.5660 6.8544 1.9126]
4	[211.4064 10.8630 6.8498 1.8618]	
4	[211.4557 10.7742 6.8650 1.8713]	
5	[211.6667 8.5542 7.1380 2.8147]	[211.6554 9.1785 7.2084 2.7585]
5	[211.6655 9.0870 7.1592 2.7632]	
5	[211.6264 8.5542 7.2227 2.7359]	

**Table 7:** The objective LEEMs comparison before and after the proposed lighting control.

Image Source	Lighting Control Method	*OM_SLDD_*	*OM_CDDDC_*	*OM_RCDDC_*	*OM_EBDDC_*
	Initial lighting (no control)	211.0342	15.1090	6.7809	2.0575
Figure 10a	Single LEEM control method	211.6236	9.3514	7.2295	2.7762
	Multiple LEEMs control method	211.6565	9.2626	7.1573	2.8662

**Table 8:** The subjective evaluation results of the lighting effect evaluation degree before and after the application of the intelligent lighting control.

Subject ID	Before Intelligent Lighting Control				After Intelligent Lighting Control			
	*SM_SLDD_*	*SM_CDDDC_*	*SM_RCDDC_*	*SM_EBDDC_*	*SM_SLDD_*	*SM_CDDDC_*	*SM_RCDDC_*	*SM_EBDDC_*
1	1	2	1	1	5	4	5	5
2	1	1	1	2	5	4	5	4
3	1	2	1	2	4	5	5	5
4	2	1	1	1	5	4	5	5
5	2	1	2	1	4	4	5	4
6	1	1	1	2	4	5	4	4
7	1	2	2	1	5	4	4	4
8	2	2	2	1	4	4	4	4

**Table 9:** The comparisons of the visual fatigue degree and the reading error rate before and after the application of the proposed intelligent lighting system.

Subject ID	Before Intelligent Lighting Control		After Intelligent Lighting Control	
	Visual Fatigue Index	Reading Error Rate Index	Visual Fatigue Index	Reading Error Rate Index
1	3	9.8%	2	5.4%
2	3	8.3%	1	4.5%
3	3	8.7%	2	4.2%
4	3	7.8%	2	4.9%
5	4	10.1%	1	5.3%
6	4	9.8%	1	6.2%
7	4	10.3%	2	4.1%
8	3	8.7%	1	4.3%
